# Supplementary material for: Modelling Skylarks (Alauda arvensis) to Predict Impacts of Changes in Land Management and Policy: Development and Testing of an Agent-Based Model
Source: PLoS One. 2013 Jun 6;8(6):e65803. doi: 10.1371/journal.pone.0065803 (PMC3675089; doi:10.1371/journal.pone.0065803)
Supplement: Supporting Information S4 — The skylark ODdox as a zipped archive. (ZIP) [file pone.0065803.s004.zip › Skylark_ODdox/class_pre_fledgeling__struct.html]

ALMaSS Skylark ODdox: PreFledgeling\_struct Class Reference


ALMaSS Skylark ODdox
 2.0


- Main Page
- Related Pages
- Classes
- Files

- Class List
- Class Index
- Class Hierarchy
- Class Members

Public Attributes

PreFledgeling\_struct Class Reference

`#include <skylarks_all.h>`

List of all members.

|  |  |
| --- | --- |
| Public Attributes | |
| int | age |
| Skylark\_Male \* | Dad |
| bool | sex |
| double | size |
| Public Attributes inherited from Skylark\_struct | |
| int | bx |
| int | by |
| Landscape \* | L |
| int | mh |
| SkTerritories \* | Terrs |
| int | x |
| int | y |

|  |  |
| --- | --- |
| Additional Inherited Members | |
| Public Member Functions inherited from Skylark\_struct | |
| virtual | ~Skylark\_struct () |

---

## Member Data Documentation

int PreFledgeling\_struct::age

Referenced by Skylark\_Population\_Manager::CreateObjects(), and Skylark\_Nestling::st\_Maturing().

Skylark\_Male\* PreFledgeling\_struct::Dad

Referenced by Skylark\_Population\_Manager::CreateObjects(), and Skylark\_Nestling::st\_Maturing().

bool PreFledgeling\_struct::sex

Referenced by Skylark\_Population\_Manager::CreateObjects(), and Skylark\_Nestling::st\_Maturing().

double PreFledgeling\_struct::size

Referenced by Skylark\_Population\_Manager::CreateObjects(), and Skylark\_Nestling::st\_Maturing().

---

The documentation for this class was generated from the following file:

- skylarks\_all.h


- PreFledgeling\_struct
- Generated on Thu Jan 10 2013 13:15:37 for ALMaSS Skylark ODdox by
   1.8.1.1
